# Outcomes of Minimally Invasive Mitral Valve Surgery Using a Multidisciplinary Team Approach: A Single-Center Experience

**DOI:** 10.3390/jpm16010044

**Published:** 2026-01-09

**Authors:** Nicolas Mourad, Durr Al-Hakim, Rosalind Groenewoud, Bader Al-Zeer, Neil Wu, Amy Myring, Julie Nakahara, David Wood, Travis Schisler, Richard C. Cook

**Affiliations:** 1 Faculty of Medicine, University of British Columbia, Vancouver, BC V6T 1Z4, Canada; nmourad1@student.ubc.ca (N.M.); durra@student.ubc.ca (D.A.-H.); bzeer@student.ubc.ca (B.A.-Z.); 2 Department of Cardiac Surgery, University of British Columbia, Vancouver, BC V6T 1Z4, Canada; rhmg@student.ubc.ca (R.G.); neilrl.wu@mail.utoronto.ca (N.W.); amy.myring@vch.ca (A.M.); julie.nakahara@vch.ca (J.N.); 3 Department of Cardiology, University of British Columbia, Vancouver, BC V6T 1Z4, Canada; david.wood@vch.ca; 4 Department of Anesthesiology, University of British Columbia, Vancouver, BC V6T 1Z4, Canada

**Keywords:** multidisciplinary heart team, paravertebral catheter, enhanced recovery after surgery, percutaneous cannulation, COR-KNOT^®^ DEVICE, clinical outcomes

## Abstract

**Background**: The advantage of employing multidisciplinary heart teams (MDHT) for the selection process of minimally invasive (MIS) mitral valve repair (MVr) and mitral valve replacement (MVR) has been previously substantiated. Here, we outline the contributions each member of the MDHT at our institution made during the intra-operative and peri-operative periods and describe their impacts on short-term outcomes. **Patients and Methods**: This is a single-center retrospective review of all 278 adult patients who underwent MIS MVR or MVr by a single surgeon at our institution between 2006 and 2023. The repair’s efficacy was assessed intraoperatively and at 1 year post-operation. The surgical technique involved a mini-thoracotomy and valve repair or replacement. Outcomes included post-operative mortality, complications, operative time, repair success rate, hospital length of stay (LOS), and post-operative ejection fraction. There was no control group, as all patients undergoing MIS MVR/MVr were treated within an MDHT model. **Results:** Delivery of regional anesthesia via paravertebral catheter (PVC) was associated with a statistically significant shorter hospital LOS (6.52 vs. 7.81 days, *p* = 0.028). Enhanced Recovery After Surgery (ERAS) implementation by nurses was associated with a potentially clinically important, although not statistically significant, reduction in LOS (6.7 vs. 10.1 days, *p* = 0.168). Introduction of the COR-KNOT^®^ DEVICE for securing annuloplasty sutures was associated with a statistically significant reduction in operative time (288 vs. 326 min, *p* < 0.001). Percutaneous cannulation, proctored by interventional cardiology in 2019, was associated with a decrease in lymphocele rate from 6.2% before 2019 to 0% after 2019. **Conclusions:** Initiatives implemented by our MDHT were associated with reduced post-operative LOS, shorter operative times, and lower incidence of post-operative complications.

## 1. Introduction

Mitral valve surgical intervention, utilizing a minimally invasive surgical (MIS) approach via right mini-thoracotomy, has emerged as a well-documented and efficacious strategy [1,2]. This approach has been associated with an expedited post-operative recovery trajectory and a reduced incidence of complications when compared to the conventional median sternotomy approach. Previous studies have suggested that MIS mitral valve surgery is correlated with shorter hospital stays, reduced post-operative pain, less post-operative bleeding, and superior cosmesis relative to conventional sternotomy [3,4].

While conventional sternotomy affords the surgeon unobstructed access to the heart, vessels, and mitral valve, thereby ensuring the safe execution of the procedure, the most prevalent minimally invasive approach for mitral valve repairs is the right thoracotomy through a minor incision on the right side of the chest in the 3rd or 4th intercostal space. Minimally invasive cardiac surgery necessitates the utilization of elongated shafted instruments through a constricted opening [5]. This presents a steep learning curve and is challenging to master [6,7].

The intricate nature of this type of procedure demands a comprehensive skill set from the entire healthcare team. The surgeon is not the only one who approaches the operation differently, but other members of the heart team as well. The pivotal role and benefits of a multidisciplinary heart team (MDHT) have been previously delineated in the context of treatment decisions for patients, demonstrating improved outcomes in the case of mitral valve replacement (MVR) and mitral valve repair (MVr) [8,9,10]. The MDHT at our institution included cardiac surgeons, interventional cardiologists, anesthesiologists, perfusionists, operating room (OR) nurses, and nurses specializing in ERAS.

While the advantage of employing MDHTs for the treatment selection process for MVR and MVr has been substantiated by prior studies, there is a dearth of research on the skillset each member contributes during the intra-operative and peri-operative periods and how that impacts patient outcomes. Thus, this retrospective single-center report has two primary aims: (1) to report preoperative and 1 year echocardiography outcomes for minimally invasive MVR and MVr procedures performed at our center within the framework of an MDHT and (2) to describe the specific contributions made by the various members of the MDHT and the impact of those contributions on patient outcomes.

## 2. Patients and Methods

### 2.1. Patient Population and Study Design

The patient population for this study comprised all consecutive adult patients who underwent an MIS MVR or MVr at our institution from January 2006 to December 2023 by a single surgeon, all of whom were treated within an MDHT model. There was no comparison to patients undergoing sternotomy-based MVR/MVr, as these patients were not treated using an MDHT model. Similarly, there was no comparison to patients undergoing minimally invasive MVR/MVr outside an MDHT, as ALL patients at our institution who underwent minimally invasive MVR/MVr were managed within the MDHT model.

Mitral valve pathology was confirmed, and the efficacy of mitral valve repairs was evaluated intraoperatively using transesophageal echocardiography (TEE) performed by an anesthesiologist certified in echocardiography. All procedures were performed by a single surgeon (R.C.). Post-operative follow-up was conducted with transthoracic echocardiography (TTE) at 1 year. Data for follow-up TTE were collected through a review of institutional records and by contacting each patient’s home cardiologist when institutional records were not available. The inclusion criteria for patients to undergo a minimally invasive mitral procedure in the initial period were only patients with normal left ventricular ejection fraction (LVEF) and isolated P2 prolapse. However, as the experience of the surgeon and team improved, patients with more complex valve pathology, patients with less than normal LVEF, and patients who had previous cardiac surgery were accepted for MIS mitral valve surgery. Given that our center does not have access to the AtriClip device for atrial appendage ligation, and since the publication of the trial by Whitlock et al. [11] demonstrating its benefit, patients with atrial fibrillation have been excluded from undergoing MIS mitral valve surgery. Additionally, due to an observed higher rate of conversion to sternotomy or failed attempted repair in patients with pectus excavatum or an intrathoracic anterior–posterior dimension of <10 cm, patients with these anatomic features are no longer offered MIS mitral valve surgery. Relatively stable contraindications included the following: (1) very severe pulmonary hypertension; (2) severe lung disease precluding single lung ventilation; (3) very low (<30%) LVEF; and (4) severe peripheral vascular disease. This retrospective study was approved by the UBC Clinical Research Ethics Board (H25-01934) on 17 June 2025, and all procedures followed were in accordance with their ethical guidelines for the use, de-identification, and storage of patient data.

### 2.2. Surgical Technique

The surgical technique employed involved the placement of a paravertebral catheter at the outset of some cases. This was followed by evaluation of the heart and mitral valve by TEE. Early in the experience, the femoral artery and vein were cannulated through a 2–3 cm incision in the groin. After 2019, the right femoral artery and vein were cannulated percutaneously, with the artery closed using Pro-Glide sutures (Abbott Vascular Devices, Redwood City, CA, USA) and the pre-close technique developed by interventional cardiologists [12]. A 5–6 cm mini-thoracotomy incision was made via the 3rd or 4th intercostal space on the right side. Myocardial protection was achieved using antegrade cold blood cardioplegia initially and then with a combination of antegrade and retrograde cold blood cardioplegia. In both cases, after an initial arrest dose of ~1000 mL, intermittent doses of ~500 cc of cardioplegia were administered every 20 min. In cases where retrograde cardioplegia was used, the retrograde catheter was placed by the surgeon, through a purse-string in the right atrium, through the mini-thoracotomy incision, using TEE guidance. The mitral valve was then inspected and repaired using an annuloplasty ring with 10–15 hand-tied sutures and/or secured with the COR-KNOT^®^ device (LSI Solutions, Victor, NY, USA). In cases of MVr, the repair was completed using a variety of strategies, including leaflet resection, cleft closure, leaflet and annulus decalcification, neochord placement, and/or annuloplasty ring placement. In cases of replacement, a prosthetic valve was placed with 15–20 sutures and secured with hand-tied sutures or using the COR-KNOT^®^ device. Following the discontinuation of cardiopulmonary bypass, TEE was performed to ensure complete de-airing of the left atrium and ventricle and to evaluate valve function and ejection fraction. The pericardium was then closed with 2–3 interrupted sutures. Peripheral pulses were checked post-operatively. All patients were transferred to the intensive care unit while intubated following surgery and were monitored in the usual fashion. Goal-directed treatment was performed with the application of vasoactive drugs, blood products, and fluid to maintain stable hemodynamics.

### 2.3. Outcomes/Impact of MDHT Roles and Contributions

Operative reports, discharge summaries, angiograms, echocardiograms, and patient consultations were retrospectively reviewed to measure procedural safety and efficacy. Echocardiogram laboratories, cardiologists, and general practitioners were also contacted to collect follow-up transthoracic echocardiogram reports for durability assessment at 1 year post-operation. Outcomes reported included post-operative mortality and intra-operative complications such as conversion to sternotomy, operative times, rate of paravertebral cannulation, rate of percutaneous cannulation, post-operative length of stay, post-operative ejection fraction, and rate of unsuccessful repair—defined as an attempted repair with greater than mild residual mitral regurgitation (MR) and/or requiring subsequent MVR. Post-operative complications, including infection, stroke, re-opening for bleeding, post-operative atrial fibrillation, acute renal failure (ARF) defined as an increase of 1.5× from baseline creatinine, and groin lymphocele, were also recorded.

### 2.4. Statistical Analysis

All data are represented as n (%) for categorical variables. For continuous variables, if they follow a normal distribution, they are presented as mean ± standard deviation (SD). For non-normally distributed continuous variables, they are presented as the median along with the interquartile range (IQR). Differences in operative, post-operative, and follow-up TTE parameters were analyzed using a one-way ANOVA, and unequal variance was accounted for via Welch tests or repeated measures logistic regression. To explore an appropriate statistical model, the distribution of the outcome was checked from the histogram and the description. Since both length of stay and operative time were right-skewed, the relationship between covariates and length of stay or operative time was explored using non-linear regression analysis based on logarithmic transformation of the outcomes. Multivariable regression analysis was conducted based on Akaike information criteria (AIC). All analyses were conducted using SPSS v28.0.0.1 (IBM SPSS Statistics, Armonk, NY, USA: IBM Corp). A *p*-value of less than 0.05 was considered statistically significant, indicating that the observed results are unlikely to have occurred by chance alone under the null hypothesis.

## 3. Results

### 3.1. Patient Cohort

Out of a total of 799 patients who underwent mitral valve procedures by the single surgeon between January 2006 and December 2023, 521 (65.2%) were operated on using a traditional sternotomy approach, and 278 (34.8%) patients underwent a minimally invasive MVr or MVR at our institution. Baseline clinical characteristics are summarized in Table 1, and preoperative echocardiographic data and operative details are summarized in Table 2. The median age was 62 years (IQR, 54–71), and 27% were women. 79 (28%) presented with New York Heart Association class III/IV heart failure.

### 3.2. Peri-Operative Outcomes

Peri-operative outcomes are summarized in Table 3. Overall, 30 day mortality was 0.36% (1 patient). This was a patient with pre-operative endocarditis who died after a major intracranial hemorrhage on post-operative day 6. Nine patients (3.2%) developed acute renal failure, 6 (2.2%) patients required reoperation for bleeding, 3 (1%) had a major wound infection, and 2 (0.7%) patients had cerebrovascular accidents.

MVR by the minimally invasive approach was performed in 29 patients, and MVr in 249 patients. Fifteen (5.4%) patients had a failed attempted repair with greater than mild MR or requiring an MVR, with a successful repair rate of 94.6%. Immediate post-repair or post-replacement transesophageal echocardiography demonstrated a mean gradient across the valve of 2.11 ± 1.08 mmHg (n = 263) and 7 (2.5%) cases of dynamic systolic anterior motion (SAM).

Six (2.2%) patients required a sternotomy due to either difficult exposure or complex valve pathology. Finally, 4 (1.4%) patients underwent a concomitant maze procedure, and 1 (0.36%) underwent a concomitant tricuspid valve repair. Annuloplasty rings were used in 241 (95.1%) repairs, predominantly CE Physio II, with most repairs involving isolated P2 prolapse, as well as anterior leaflet (6.6%) or commissural involvement (6.6%).

Median operation room (OR) time was 299 (IQR, 266–340) min, cardiopulmonary bypass (CBP) time was 175.5 (IQR, 148.4–199.3) min, cross clamp (XC) time was 126.5 (IQR, 105.8–153.3) min, and post-operative hospital length of stay (LOS) was 6 days (IQR, 5–7).

### 3.3. Impact of MDHT Roles and Contributions

**Figure 1:**
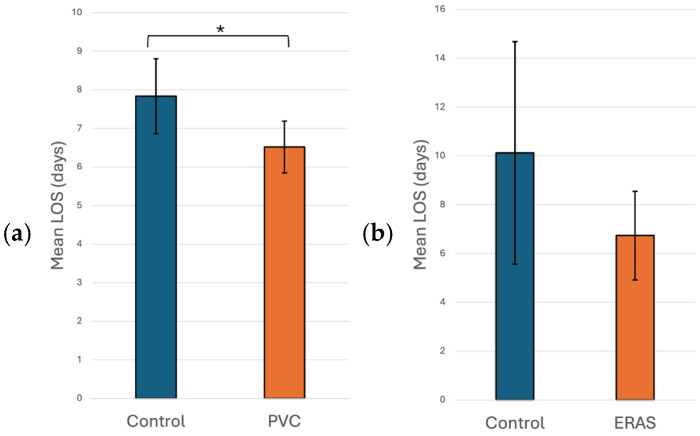
(**a**) Hospital LOS with PVC vs. without PVC (control) and (**b**) hospital LOS with ERAS vs. without ERAS (control). * = *p* < 0.05, PVC = Paravertebral Catheter, LOS = Length of Stay, ERAS = Enhanced Recovery After Surgery.

**Figure 2:**
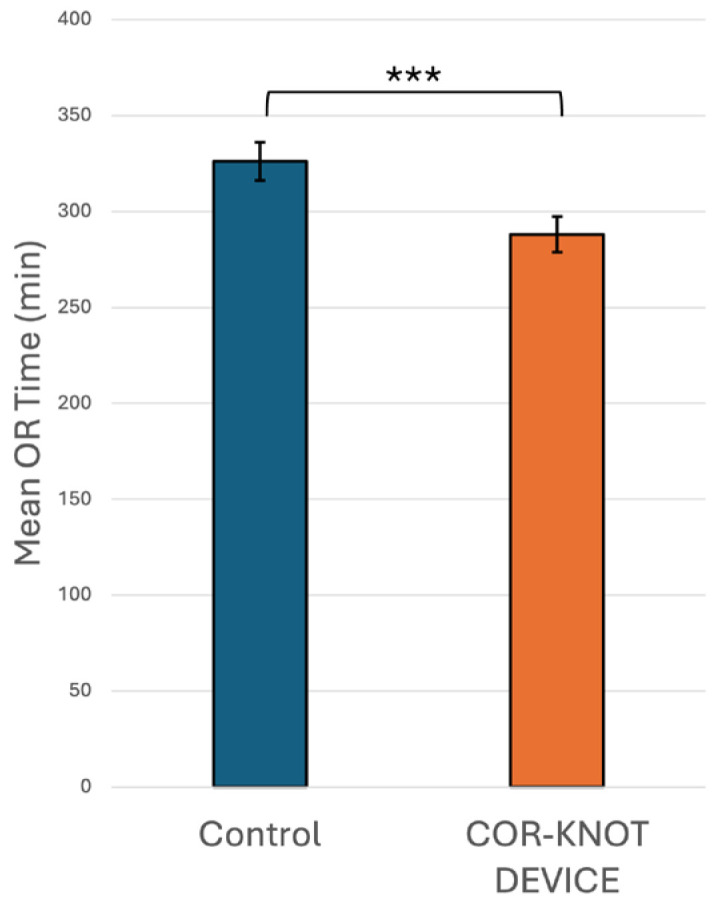
OR time with COR-KNOT^®^ DEVICE vs. without COR-KNOT^®^ DEVICE (control). *** = *p* < 0.001, OR = Operating Room.

1. Anaesthesiology: Many aspects of mini-thoracotomy MVR/MVr require additional skills on the part of our anesthesia colleagues. We use a bronchial blocker to isolate the right lung and rely on the TEE skills of the cardiac anesthesiologist to show us the mitral valve pathology and place a retrograde cardioplegia cannula. At our institution, they also perform percutaneous right internal jugular cannulation for superior vena cava drainage. However, the greatest impact that our anesthesia colleagues have made is in the management of post-operative pain, which has been a source of major morbidity following mini-thoracotomy mitral valve surgery. In an attempt to mitigate this, our cardiac anesthesia group started to use regional anesthesia through the placement of a paravertebral catheter (PVC). The PVC is inserted on the side of the surgery before the induction of anesthesia. This procedure was performed by an anesthesiologist trained in ultrasound or landmark-based placement with the patient in the sitting position. A total of 135 (48.6%) patients received PVC-based regional anesthesia, which was associated with a statistically significant shorter hospital LOS compared to no PVC use (6.52 vs. 7.81 days, *p* = 0.028), as seen in Figure 1a. This was likely due to reduced pain, as patients subjectively appeared more comfortable and mobile when a PVC was used. Unfortunately, pain scores were not available to quantify the impact of PVC use on pain.
  In the univariate non-linear regression model, the expected LOS for patients in the PVC group was about 11.8% shorter compared with patients in the control group (*p* = 0.028). After adjusting for significant risk factors (i.e., age, sex, and ejection fraction) in the multiple regression analysis, patients who had a PVC still had a significantly shorter LOS than control group patients, with the expected LOS for patients in the PVC group being about 16.9% shorter compared with patients in the control group (*p* = 0.019; Table 4).
2. Interventional Cardiology: Prior to 2019, all patients undergoing minimally invasive MVR/MVr were cannulated femorally through a small cut-down in the groin for direct cannulation of the femoral artery and vein. Using this technique, we noticed that some patients developed persistent lymphoceles at this site. Under the instruction and supervision of our interventional cardiology colleagues, we switched to percutaneous cannulation of the femoral artery and vein, using the “preclose” technique for the artery as developed for transcatheter aortic valve replacement. Prior to the implementation of percutaneous cannulation, 11 patients developed post-operative lymphoceles (11/178 patients, 6.2%). In comparison, 100 (36.0%) cases were carried out using percutaneous cannulation, with no incidence of lymphoceles in this group of patients (0/100 patients, 0%).
3. Operative Nursing: The operative nursing team has had to adapt to using a completely different set of instruments, as regular instruments cannot be used through a small mini-thoracotomy incision. As such, long-shafted instruments are used instead, which requires some adaptation on the part of the operative nursing team. One particularly difficult maneuver with long-shafted instruments is the tying of knots. This can be time-consuming, with an increased risk of air knots and/or broken sutures. At our institution, the operative nursing team is in control of all OR instrumentation purchases. With their assistance, we were able to bring the COR-KNOT^®^ DEVICE to our institution to eliminate the need to tie knots manually. In the 132 (47.5%) patients in whom COR-KNOTs were used, there was a statistically significant 38 min decrease in OR time when compared to patients who had knots tied with long-shafted instruments (288 vs. 326 min, *p* < 0.001), as seen in Figure 2.
  In the univariate non-linear regression model, the expected operative time for patients in the COR-KNOT^®^ DEVICE group was about 12.6% shorter compared with patients in the control group (*p* < 0.001). After adjusting for significant risk factors (i.e., sex, BMI, and surgery time) in multiple regression analysis, patients who had a COR-KNOT^®^ DEVICE still had a significantly shorter operative time than control group patients, with the expected operative time for patients in the COR-KNOT^®^ DEVICE group being about 6.5% shorter compared with patients in the control group (*p* = 0.022; Table 4).
4. Post-Operative Nursing: At our institution, the development of Enhanced Recovery After Surgery (ERAS) pathways following surgery is developed by our nursing colleagues. After having implemented ERAS guidelines in 38 (13.7%) patients, we noticed a non-statistically significant decrease in hospital LOS when compared to no ERAS pathway (6.7 vs. 10.1 days, *p* = 0.174), as seen in Figure 1b.

**Table 4:** Comparison of outcomes between treatments.

Outcomes	Univariate Analysis		Adjusted for Potential Risk Factors	
	Estimate (95% CI)	*p* Value	Estimate (95% CI)	*p* Value
**Hospital LOS (days)** ^†^				
PVC (yes vs. no)	−0.126 (−0.238, −0.014)	0.028	−0.185 (−0.340, −0.031)	0.019
Age, years	−0.002 (−0.004, 0.001)	0.06	0.009 (0.004, 0.013)	<0.001
Female sex	−0.083 (−0.138, −0.028)	0.003	0.152 (0.031, 0.274)	0.014
EF (%)	−0.019 (−0.029, −0.009)	<0.001	−0.013 (−0.020, −0.005)	0.001
Surgery year, years	−0.008 (−0.026, 0.011)	0.42	0.004 (−0.016, 0.023)	0.70
**Operation time (minutes)** ^‡^				
COR-KNOT^®^ DEVICE (yes vs. no)	−0.135 (−0.182, −0.089)	<0.001	−0.067 (−0.124, −0.010)	0.022
Female sex	−0.083 (−0.138, −0.028)	0.003	−0.063 (−0.114, −0.012)	0.016
BMI (kg/m^2^)	0.011 (0.006, 0.017)	<0.001	0.009 (0.004, 0.014)	<0.001
Surgery year, years	−0.019 (−0.025, −0.014)	<0.001	−0.013 (−0.020, −0.006)	<0.001

LOS: Length of stay in hospital; PVC: paravertebral catheter; CI: confidence interval. ^†^ Estimate: log of hospital LOS; for PVC group compared with no PVC group, (exp(−0.126) − 1) × 100 = 11.8% shorter in univariate analysis, and (exp(−0.185) − 1) × 100 = 16.9% shorter in multivariable analysis. ^‡^ Estimate: log of operative time; for COR-KNOT^®^ DEVICE group compared with no COR-KNOT^®^ DEVICE group, (exp(−0.135) − 1) × 100 = 12.6% shorter in univariate analysis, and (exp(−0.067) − 1) × 100 = 6.5% shorter in multivariable analysis.

### 3.4. One-Year Echocardiography Follow-Up

**Figure 3:**
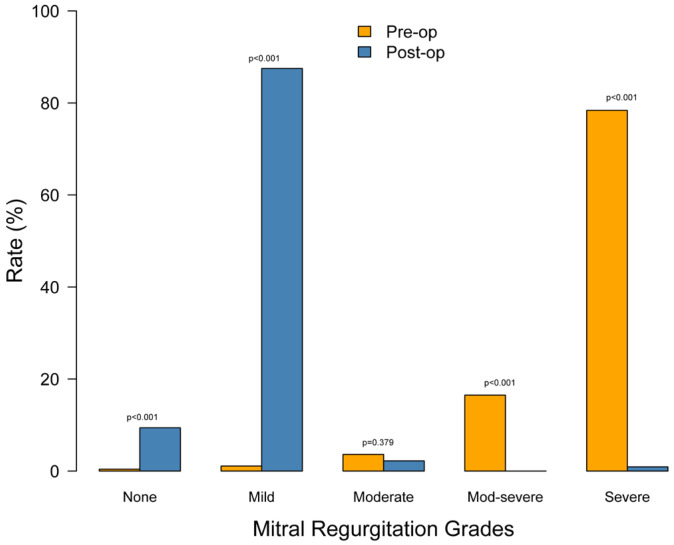
Incidence of mitral regurgitation preoperatively and at 1 year post-operatively.

We conducted a follow-up appointment one year post-operatively, which included echocardiography for evaluating MR. Table 5 and Figure 3 compare pre-operative to post-operative echocardiogram MR findings, showing a substantial improvement from 94.5% of patients having moderate-severe or severe MR pre-operatively to 93% of patients having none or trivial MR 1 year post-operatively.

## 4. Discussion

Our study’s findings highlight the possibility of achieving excellent results with MIS MVR and MVr procedures, with extremely low peri-operative mortality and morbidity and a successful repair rate of 94.6%. This high success rate is indicative of the potential for the minimally invasive approach to deliver positive patient outcomes.

However, the success of these procedures is not solely dependent on surgical expertise, but on the multidisciplinary team, which integrates adjunctive intra-operative and peri-operative interventions that optimize patient care and recovery. Performing mitral valve surgery through a right mini-thoracotomy approach requires a different set of skills from many members of the MDHT, with each patient presenting unique challenges. For example, some patients will require cannulation of both the right internal jugular vein and a femoral vein, whereas others will only require femoral venous cannulation. In some patients, regional anesthesia can allow for faster mobilization, but in some patients, placement of a paravertebral catheter is not possible because of their particular anatomic features. In addition, some patients can be ventilated easily with a bronchial blocker in the right main bronchus, whereas others will need a double-lumen endotracheal tube for right lung isolation. Hence, we feel that patients undergoing MIS MV surgery require the personalized approach allowed for by an MDHT model to ensure optimal outcomes.

Although the lack of a control group makes it impossible to assign a causative relationship between the MDHT model and the results achieved, we feel that three key approaches have significantly changed the practice of MIS mitral valvular surgery at our institution. The first is the delivery of regional anesthesia with paravertebral cannulas. Our team had previously conducted a study at our institution showing statistically significant reductions in post-operative hospital LOS, time to extubation, and elective oxycodone consumption in patients who received PVC-based regional anesthesia compared with the control group [13]. Previous studies have also demonstrated that PVC-based regional anesthesia in patients undergoing thoracic surgery has a relatively low side effect profile, is associated with hemodynamic and respiratory stability, and decreases LOS, with comparable or better pain control compared with traditional thoracic epidural anesthesia (TEA) [14,15,16,17]. Furthermore, PVC-based regional anesthesia can spare normal respiratory and sympathetic functions on the contralateral side with unilateral blocks, thereby reducing the incidence of hypotension, pulmonary complications, urinary retention, and other complications seen with TEA [18,19].

The second approach that has contributed to the advancement of minimally invasive mitral valvular surgery at our institution was percutaneous femoral arterial and venous cannulation [20]. To limit the thoracotomy incision size, most surgeons perform peripheral/groin cannulation for CPB through a groin incision and direct cannulation of the vessels. This procedure is safe and offers the advantage of direct cannulation of the vessels, which may minimize iatrogenic cannulation-related complications. However, some patients may return several days after surgery due to complications related to groin cannulation, such as lymphocele formation, which may occur due to injury of the lymph vessels at the time of cannulation [21,22], or wound infection. These complications may necessitate readmission and may require weeks of wound management. Since we started performing percutaneous cannulation more often in 2019, we have observed no instances of post-operative lymphoceles, thereby reducing the lymphocele rate from 6.2% to 0%. This finding is consistent with other centers that have been performing MIS MV surgery with percutaneous cannulation [23].

The third approach is the use of the COR-KNOT^®^ DEVICE. Long operative times, such as cardiopulmonary bypass (CPB) and aortic cross clamp (AXC) time, may lead to a higher risk of multiple organ dysfunction syndrome and adverse outcomes [24]. A consistent finding in almost all the published literature of MVr through a right mini-thoracotomy is of increased operative times compared with sternotomy, especially at the start of the learning curve [7,25]. Thus, we employed this automated knot fastener device, which has been previously shown to lower operative times in patients undergoing MIS cardiac surgery [26,27] and was associated with an average reduction in OR time of 38 min in our experience.

The successful implementation of these adjunctive interventions requires a well-coordinated MDHT. Though the cardiac surgeon is central to performing minimally invasive mitral valve surgery, our MDHT appeared to be associated with improvements in surgical and patient outcomes, with each discipline bringing its unique expertise to the table. At our institution, the MDHT includes cardiac anesthesiologists, interventional cardiologists, perfusionists, nurses, nursing leadership, ERAS coordinators, and cardiac surgeons. Other healthcare professionals, such as physiotherapists, dietitians, and pharmacologists, are involved in the care of all of our cardiac surgical patients but did not make changes specific to the care of patients undergoing minimally invasive surgery.

The many contributions of the cardiac anesthesiologists have already been delineated. However, in our view, the positive impact of regional anesthesia delivered by PVC cannot be overstated because of the effect it has had on enhancing patient comfort and recovery, as well as improving healthcare resource utilization by reducing post-operative length of stay [28].

Interventional cardiologists brought their unique expertise to the MDHT by teaching the surgeons how to perform pre-closure percutaneous cannulation in femoral arteries and veins. They ensured the procedure was conducted safely, and their expertise has been crucial in minimizing post-operative complications such as seromas, thereby enhancing overall patient outcomes [29,30].

Our perfusionists have demonstrated their expertise in managing the challenges of peripheral cannulation and alternative myocardial protection techniques such as ventricular fibrillation (VF) arrest. These techniques are particularly important in minimally invasive mitral valve surgery, where venous drainage can be much more challenging

Preoperatively, our nurses provide education and resources to the patient on their surgical journey. Intraoperatively, they have become proficient in positioning the patient for a right mini-thoracotomy approach, which requires different positioning on the table, different preparation (e.g., defibrillator pads), and other precautions to prevent post-operative complications such as ulnar nerve palsy or brachial plexus injury [31]. They have also familiarized themselves with the new instruments used in MIS, including long-shafted instruments and the COR-KNOT^®^ DEVICE.

In December 2020, our MDHT implemented ERAS peri-operative protocols with this population. ERAS is a patient-centered, evidence-based approach that includes several peri-operative strategies to improve recovery, including early mobilization and line/drain removal, aggressive pain and nausea management, and better patient education. These protocols are crucial for improving patient experience and outcomes, such as shortening hospital LOS and preventing post-operative complications, including infections, ARF, cerebrovascular accidents, and bleeding [32]. Other centers performing MIS MV surgery that have implemented ERAS pathways have reported improved patient outcomes [33]. In a small initial group of patients, we observed a non-statistically but likely clinically important reduction in hospital LOS of 3.4 days when ERAS guidelines were implemented consistently in patients undergoing MIS mitral valve surgery.

### Limitations

While we recognize that our study only included one surgeon, we believe that the excellent results observed are due in large part to the multidisciplinary approach used in our minimally invasive MV surgery program. The contributions made by cardiac anesthesiologists, interventional cardiologists, nurses, and perfusionists were all critical for ensuring the success, growth, and development of our program. As a result, we believe that having an MDHT approach would also be beneficial in a multi-surgeon practice.

Other limitations of this study include the retrospective, single-center, single-surgeon study design, all of which limit the generalizability of the findings, in addition to the inherent risk of patient selection bias with this type of study.

Over time, more complex valve pathology and lower LVEF cases were accepted as experience increased. This temporal heterogeneity makes the study population less uniform and may confound the interpretation of short-term outcomes such as operative time, complications, and LOS.

The absence of a non-MDHT comparator group was another limitation of this study. However, there were no comparable “control” patients available at our institution, as ALL patients undergoing minimally invasive MV surgery at our institution were treated within the MDHT model.

## 5. Conclusions

Patients at our institution who underwent minimally invasive MV surgery using an MDHT model experienced excellent outcomes with only one peri-operative mortality (0.36%) and a successful repair rate of 94.6%. The contributions of members of the MDHT included the introduction of regional anesthesia, percutaneous femoral arterial and venous cannulation, the use of the COR-KNOT^®^ DEVICE, and the ERAS protocol, which we feel have advanced the practice of minimally invasive mitral valve surgery at our institution. Further research is needed to validate these findings and explore their implications in different clinical settings.

## Figures and Tables

**Table 1:** Baseline clinical characteristics.

Variables	Summary (Total n = 278)
Age in years, median (IQR)	62.0 (54.0–71.0)
Male gender, n (%)	204 (73.3)
BMI, mean (SD)	25.6 (±4.4)
EF%, mean (SD)	58.0 (±7.5)
Dialysis, n (%)	2 (0.7)
Hypertension, n (%)	113 (40.6)
Endocarditis, n (%)	21 (7.6)
Lung disease, n (%)	34 (12.2)
On immunosuppression, n (%)	7 (2.5)
Peripheral vascular disease, n (%)	10 (3.6)
Cerebrovascular disease, n (%)	8 (2.9)
Previous CABG, n (%)	2 (0.7)
Previous valve surgery, n (%)	4 (1.4)
Previous PCI, n (%)	16 (5.8)
Previous MI, 10 (%)	5 (1.8)
Angina, n (%)	49 (17.6)
Heart failure (NYHA Class), n (%)	256 (92.1)
Type I	66 (25.8)
Type II	111 (43.3)
Type III	59 (23.1)
Type IV	20 (7.8)
Resuscitation, n (%)	0 (0.0)
Arrhythmia, n (%)	79 (28.4)
Inotropes, n (%)	187 (67.3)

BMI = body mass index; EF = ejection fraction; CABG = coronary artery bypass graft; PCI = percutaneous coronary intervention; MI = myocardial infarction; NYHA = New York Heart Association.

**Table 2:** Preoperative echocardiographic data and operative details.

Variables	Summary (Total n = 278)
Aortic stenosis, n (%)	2 (0.7)
Mitral stenosis, n (%)	18 (6.5)
Aortic insufficiency, n (%)	40 (14.4)
Mild	38 (32.5)
Moderate	2 (5.0)
Moderate-Severe	0 (0)
Severe	0 (0)
Mitral regurgitation, n (%)	277 (99.6)
Mild	3 (1.1)
Moderate	10 (3.6)
Moderate-Severe	46 (16.6)
Severe	218 (78.7)
Tricuspid regurgitation, n (%)	243 (87.4)
Mild	198 (71.2)
Moderate	41 (16.9)
Moderate-Severe	2 (0.8)
Severe	2 (0.8)
Procedure status, n (%)	
Elective	246 (88.5)
Urgent	32 (11.5)
Operation duration in minutes, median (IQR)	299.0 (266.0–340.0)
Cardiopulmonary bypass, median (IQR)	175.5 (148.4–199.3)
Aortic cross clamp, median (IQR)	126.5 (105.8–153.3)
Repair type, n (%)	
Neochord use	100 (41.0)
Isolated P2 repair	94 (38.5)
Anterior leaflet repair	16 (6.6)
Commissure repair	16 (6.6)
Ring annuloplasty	
CE Physio II	232 (95.1)
St. Jude Seguin	9 (3.7)
No ring	3 (1.2)
PVC use, n (%)	
No	143 (51.4)
Yes	135 (48.6)
ERAS, n (%)	
No	240 (86.3)
Yes	38 (13.7)
COR-KNOT^®^ DEVICE, n (%)	
No	146 (52.5)
Yes	132 (47.5)
Percutaneous cannulation, n (%)	
No	178 (64.0)
Yes	100 (36.0)

PVC = paravertebral catheter; ERAS = Enhanced Recovery After Surgery.

**Table 3:** Perioperative outcomes.

Variables	Summary (Total n = 278)
Acute Renal Failure, n (%)	9 (3.2)
Reoperation for Bleeding, n (%)	6 (2.2)
Major Wound Infection, n (%)	3 (1.1)
Cerebrovascular Accidents, n (%)	2 (0.7)
Red Blood Cell Transfusion, n (%)	33 (11.9)
Lymphocele, n (%)	11 (4.0)
Post-operative Mortality, n (%)	1 (0.4)
Operation time, median (IQR)	299.0 (266.0, 340.0)
Hospital Length of Stay in days, median (IQR)	6.0 (5.0–7.0)

**Table 5:** Echocardiogram mitral regurgitation findings pre-operatively and at 1 year post-operatively.

Grade	Pre-Op (n = 278)	Post-Op (n = 224)	*p*-Value
None	1 (0.4%)	21 (9.4%)	**<0.001**
Mild	3 (1.1%)	196 (87.5%)	**<0.001**
Moderate	10 (3.6%)	5 (2.2%)	0.379
Moderate-severe	46 (16.5%)	0 (0.0%)	**<0.001**
Severe	218 (78.4%)	2 (0.9%)	**<0.001**

## Data Availability

The datasets generated during and/or analyzed during the current study are available from the corresponding author on reasonable request.
